# On the Trail of an Ancient Middle Eastern Ethnobotany: Traditional Wild Food Plants Gathered by Ormuri Speakers in Kaniguram, NW Pakistan

**DOI:** 10.3390/biology10040302

**Published:** 2021-04-06

**Authors:** Muhammad Abdul Aziz, Zahid Ullah, Mohamed Al-Fatimi, Matteo De Chiara, Renata Sõukand, Andrea Pieroni

**Affiliations:** 1 University of Gastronomic Sciences, Piazza Vittorio Emanuele II 9, 12042 Pollenzo, Bra, Italy; a.pieroni@unisg.it; 2 Center for Plant Sciences and Biodiversity, University of Swat, Kanju 19201, Pakistan; zahidtaxon@uswat.edu.pk; 3 Faculty of Pharmacy, Aden University, P.O. Box 5411 (Maalla), Aden 00967-2, Yemen; alfatimi@web.de; 4 INALCO (National Institute for Oriental Languages and Civilizations), 2 Rue de Lille, 75007 Paris, France; matteo.dechiara@inalco.fr; 5 Department of Environmental Sciences, Informatics and Statistics, Ca’ Foscari University of Venice, Via Torino 155, 30172 Venezia, Italy; renata.soukand@unive.it; 6 Department of Medical Analysis, Tishk International University, Erbil 4401, Kurdistan, Iraq

**Keywords:** ethnobotany, wild food plants, Pashtuns, Ormur people, Burki people

## Abstract

**Simple Summary:**

Wild food plants (WFPs) have played an important role in the human diet throughout history. The current study reports WFP uses among two linguistic groups, i.e., the Ormur people and Pashtuns, living in the Valley of Kaniguram, South Waziristan, Pakistan. A total of fifty-two plants were reported among the two researched groups and these plants were mostly consumed as raw snacks and vegetables. Remarkable homogeneity was observed for WFP uses among the two groups. Being an ancient diaspora, the Ormur people have retained rich traditional knowledge of WFPs and reported some important plant uses that are believed to have arrived from the near West, most likely the Middle East. The current study is an important effort to preserve the biocultural gastronomic heritage of the Ormur people that speak the moribund language; hence, it is strongly recommended that policy makers pay attention to the cultural and traditional gastronomic heritage of the community.

**Abstract:**

An ethnobotanical field study focusing on traditional wild food botanical taxa was carried out in Kaniguram, South Waziristan, Pakistan, among Ormur (or Burki or Baraki) peoples, which represent a diasporic minority group, as well as among the surrounding Pashtuns. Through sixty semi-structured interviews, fifty-two wild food plants (taxa) were recorded, and they were primarily used raw as snacks and cooked as vegetables. Comparative analysis found a remarkable overlap of the quoted plant uses between the two studied groups, which may reflect complex socio-cultural adaptations Ormur speakers faced. Ormur people retain a rich knowledge of anthropogenic weeds and the phytonyms reveal important commonalities with Persian and Kurdish phytonyms, which may indicate their possible horticultural-driven human ecological origin from the Middle East. Some novel or rare food uses of *Cirsium* *arvense*, *Nannorrhops ritchiana*, *Periploca aphylla*, *Perovskia atriplicifolia*, *Viscum album,* *Oxalis corniculata* and *Withania coagulans* were documented. Since the Ormuri language represents a moribund language, still spoken by only a few thousand speakers in NW Pakistan and Afghanistan, it is recommended that the traditional bio-cultural and gastronomical heritage of this minority group be appropriately protected and bolstered in future rural development programs.

## 1. Introduction

The first agricultural expansion during the Neolithic Revolution drove several cultures into a wide-scale transition, namely from a lifestyle of hunting and gathering to one of agriculture and settlement [1]. Human societies evolved adaptive mechanisms in order to organize the biotic and abiotic environment and to cope effectively with the changing socio-ecological circumstances across time and space [2,3], and foraging, i.e., the collection of wild food items, and especially plants, has been crucial in *both* pre-and post-Neolithic societies.

Human and plants have long-lasting relationships, and being a part of those associations, wild food plants (WFPs) have contributed greatly to the aggregate food basket and livelihoods of local communities. In the past Century, industrialization and globalization have significantly reduced the dependence of local communities on WFPs, and this in turn has impacted the traditional ecological knowledge (TEK) around these natural resources, which co-evolved for millennia, even though WFPs are still a “hidden harvest” for many rural areas, even in industrialized countries [4,5,6].

Being an ever-changing complex, the TEK has always been renegotiated, and as a result, one of the most fascinating questions in human ecology concerns the ways and processes through which TEK has changed and is still changing across time and space [2]. It is evident that each culture generates particular knowledge of the surrounding environment and this is constantly reshaped following continuous socio-ecological changes. Cultural and linguistic edges have always played an important role in articulating human perspectives on the diverse biological world [7], and, most importantly, language holds a wealth of information about nature, including plants [8]. Researchers have always taken a keen interest in understanding TEK transmission within given cultural groups [9,10,11,12,13,14], and more specifically, the cross-cultural sharing of TEK, which has led researchers to carry out cross-cultural and cross-linguistic ethnobotanical studies ([7,15,16,17,18,19,20], and references therein).

Pakistan has immense ethnic, linguistic and religious diversity. Traditional ecological knowledge (TEK) is deeply embedded in the lifeways of people, especially those who live in rural and remote mountain areas. Due to rapid socio-cultural (d)evolution, TEK held by local communities has seriously been affected, if not eroded. More importantly, TEK held by minority groups is more vulnerable to change where the speed of TEK erosion accelerates due to the possible influence of the dominant cultures. Pakistani ethnobotany has paid little attention to the protection of the biological heritage of WFPs, especially that held by minorities, and only a few studies, which includes our recent research [18,19,20], have been carried out in North-West Pakistan. Therefore, the protection and celebration of TEK held by minority groups should always be seriously considered. 

The study was carried out in the town of Kaniguram, South Waziristan Tribal District, Pakistan. We recorded WFP uses among two different linguistic groups, i.e., the autochthonous Pashtuns and Ormur people. Ormuri speakers (also called Burki or Baraki) represent an ethnic and linguistic minority group. For the last two decades, social, political and economic circumstances have seriously affected the traditional lifeways of the region; particularly, Ormuri speakers are facing huge challenges to keep their linguistic and cultural identity intact. Therefore, this study was undertaken to protect the TEK of WFPs of the studied communities, which could play a part in mobilizing policymakers to adopt certain measures for the protection of local cultural and biological heritage. The current research aimed to study the impact of linguistic affiliation on traditional ethnobotanical knowledge related to WFPs among the aforementioned groups. The specific objectives of this study were:

(a) To record phytonyms and traditional WFP uses among the two linguistic communities. 

(b) To make a cross-cultural comparison of this gathered data between the considered linguistic communities in order to understand the historical cultural adaptation processes that these groups underwent, especially that of Ormuri speakers to the dominant Pashtun culture. 

(c) To compare the gathered data with the existing Pakistani food ethnobotanical literature. 

## 2. Materials and Methods 

### 2.1. Study Area and Communities

**Figure 1:**
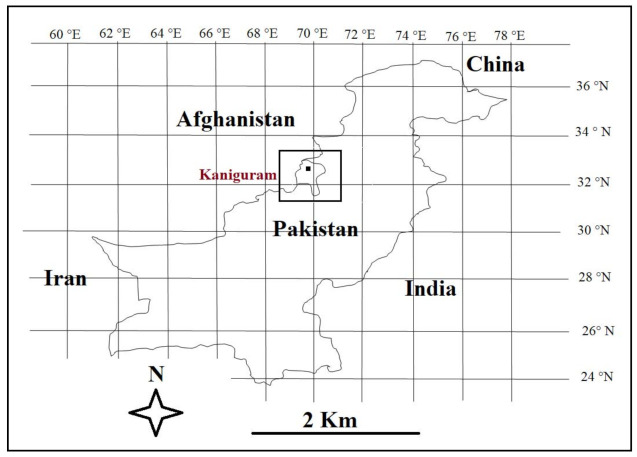
Location of the study area within Pakistan.

**Figure 2:**
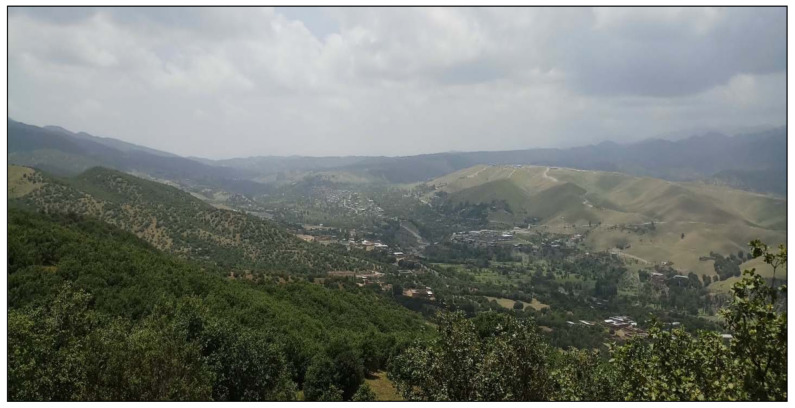
Landscape of the study area.

The study was carried out in the town of Kaniguram, located in South Waziristan, Pakistan (Figure 1). The town, which is located on a sloping hillside, is populated by Pashtuns (Mehsud tribe) and Ormuri speakers (Figure 2).

Kaniguram is principally associated with the Ormur community. In the area, Pashto is spoken as the *lingua franca* and is quite different from other places in Pashtun territory. The well-known Pashtun tribe is a large and highly self-aware ethnic group inhabiting the adjoining areas of West Pakistan and Afghanistan. The social structure of the ethnic group is mainly distinguished by certain social values. Pashtuns follow a particular code of life known as *Pakhtunwali*, which is characterized by particular customs, and a person is considered *Pashtun* if he/she embodies the characteristics expressed in the *Pakhtunwali*, which are considered necessary in order to maintain the Pashtun identity [21].

Literature on the ethnogeny of the Ormur is quite limited. Khattak [22] mentioned 10,000 speakers of the Ormuri language in the town of Kaniguram. In the past, the Ormur settled mainly in two locations: Logar in Afghanistan and Kaniguram in Pakistan [23]. Traditions relate that the Ormur may be descendants of Persians, Arabs, Kurds, or Afghans [24,25,26]. According to one hypothesis, the Ormur originated from the southern shores of the Caspian Sea in Persia and migrated to the south-eastern part of the Iranian territory relatively recently [23], while other viewpoints suggest they represent indigenous communities that occupied lands south of Hindu Kush from time immemorial. Their language, however, is the only surviving representative of a southeastern subgroup of Iranian languages [25,26,27,28]. Bellew [29] claimed that the Persian Emperor Darius Hystaspes, Governor of Egypt, conquered the Greek colonies of Barke and Kyrene in Libya and took the ancestors of today’s Ormur peoples to Egypt and then brought and settled them in a village known as Bark’e in Central Asia (Baktrian area). The colony of Barkaians would be today represented by the Ormur tribe inhabiting the villages of Baraki Barak and Baraki Rajan in the Logar district of Kabul, Afghanistan. Captain Leech [30] stated that the Ormur peoples were included in the general term *Parsiwan*, or *Tajak,* and that they could be arrived from Yemen. They could have been brought by Sultan Mahmud of Ghazni, whom they accompanied during his invasions of India in the 11th Century, and they were instrumental in the abstraction of the gates of the temple of Somnath [26,30]. Later, still in the 11th Century, during the return of Sultan Mehmud to Afghanistan, some of the Ormur soldiers that accompanied him settled in Kaniguram as they found the valley the most suitable place to live. Research also reveals their possible Zoroastrian origin, in which sacramental fire played a crucial role in their daily life [31], and this can be seen nowadays in that most of these people have adopted the professions of goldsmith and blacksmith.

According to local traditions (related by Caroe [32]), the Ormurs would be the descendants of Urmar, the son adopted by Sharkbun/Shakarbun/Sharjyun, the forefather of the Sarbanri Pashtuns or Western Afghans.

### 2.2. Vegetation and Environment

The study area is composed of a mass of rugged and complex hills and ridges. The overall area of South Waziristan comprises 6619 km^2^ and is situated at 321 24′ 50′′ N latitude and 691 42′ 06′′ E longitude, with an altitude of 1200-2100 m.a.s.l. Temperature in the area falls to 0 °C during winter in some places, with snow fall occurring at higher altitudes. The winter is extremely severe, with the coldest months from December to February. Average rainfall per annum is 6 inches, while in plain areas, the summer season is relatively hot. The natural vegetation of South Waziristan is diverse due to the irregular physiographic features of the area. At lower elevations, subtropical vegetation is dominant, whereas at higher elevations, dry temperate forests exist in a clear ecotonal zone. Kaniguram is located at a high elevation where vegetation cover is mainly composed of *Quercus* spp., and at even higher elevations, *Pinus gerardiana*, *Pinus wallichiana*, *Cedrus deodara* and *Abies pindrow* are the common botanical taxa [33]. 

### 2.3. Ethnobotanical Field Study

**Figure 3:**
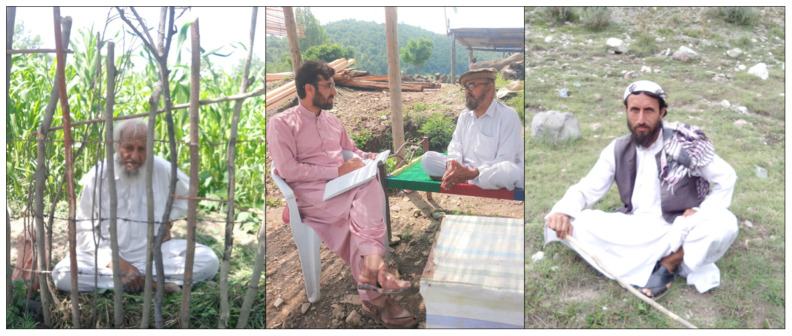
Local participants interviewed during the survey.

An ethnobotanical field study was carried out in July 2020 in the town of Kaniguram. The main purpose of the survey was to record local knowledge of WFP and mushroom uses within the two selected linguistic groups. From each of the studied groups, thirty informants were recruited through snowball techniques to participate in semi-structured interviews. Informants were selected among middle-aged and elderly inhabitants (range: 52 to 74 years old), including farmers and shepherds, which were considered the possible knowledge holders in the area (Figure 3). 

The selection criterion for the study participants was based on their strong affiliation with nature over many years. It is important to note that we were not allowed to interview female community members in order to respect their practice of Pardah (“Veil”). 

Prior to each interview, verbal consent was obtained from each of the participants and the Code of Ethics adopted by the International Society of Ethnobiology [34] was followed. Semi-structured interviews were conducted in the Pashto language. The interviews were focused on gathered WFPs used as cooked vegetables, raw in salads, as snacks, as seasoning, or for recreational teas. Specific questions were also asked concerning wild plants possibly used in dairy products or in lactic fermented foods, as well as the consumption of edible mushrooms. For each of the free listed plants recorded during the study, the local name and local food uses were documented. Additionally, qualitative ethnographic information was gathered via open-ended questions and participant observation. The quoted wild food taxa were then collected from the study area and identified by the third author using the *Flora of Pakistan* [35,36,37,38]. Voucher specimens were later deposited at the Herbarium of the Department of Botany, University of Swat, Khyber Pakhtunkhwa, Pakistan. Identification of the few wild plants for which it was not possible to collect vouchers was made on the basis of the folk name and detailed plant description only. Nomenclature followed The Plant List database [39] for each plant taxon and the Index Fungorum [40] for the mushroom taxon, while plant family assignments were consistent with the Angiosperm Phylogeny Website [41].

### 2.4. Data Analysis

Ethnobotanical taxa and their uses were compared between the two community groups through Venn diagrams, which were drafted using free software (http://bioinformatics.psb.ugent.be/webtools/Venn/ accessed on 22 March 2021). Moreover, for national data comparison, a detailed literature survey on the ethnobotany of WFPs of Pakistan was also conducted [18,19,20,42,43,44,45,46,47,48]. 

The cross-linguistic analysis was carried out using the most comprehensive folk plant reviews in Persian, Kurdish, and Yemeni Arabic [49,50,51].

## 3. Results and Discussion

### 3.1. Wild Food Plant Uses

The wild plant-based gastronomic heritage of the two linguistic groups is comprised of fifty-two botanical taxa, which includes three mushroom taxa (Table 1). 

Regarding the food categories assigned to the reported WFPs, 52% of the taxa are used as raw snacks (26 taxa) and more than two thirds are cooked as vegetables (20 taxa). In food ethnobotanies, raw snack consumption primarily emerges during the adoption of mobile pastoralism, which local communities might have co-evolved. Our findings are in line with previous ethnobotanical studies that frequently reported snacks as the dominant food category [19,20,52,53,54]. The most commonly quoted wild vegetables were *Allium spp., Amaranthus viridis*, *Lepidium draba*, *Portulaca quadrifida*, *Polygonatum verticillatum*, *Rumex dentatus*, *Urtica dioica* and an unidentified taxon, and these wild vegetables have been frequently reported in the Pakistani food ethnobotanical literature [18,19,20,42,43,44,45,46,47,48]. The majority of the recorded wild vegetables are weeds, possibly gathered in anthropogenic environments since these weeds are prevalent in various food ethnobotanies, which may be due to their broad ecological amplitude [55,56]. Researchers contend that anthropogenic environments provide an important space for WFP collection [57]. It is always advisable to focus on the gathering environment of WFPs rather than looking solely at the number of species gathered [58]. In a previous study, Ahmad et al. [48] reported the frequent use of weeds as wild vegetables by different ethnic communities in the North West Frontier Province of Pakistan. Researchers argue that weeds may be a better alternative for coping with food scarcity and achieving sustainable nutritional goals [59,60,61]. The recorded WFPs were largely herbs and aerial parts, which were the most commonly used plant parts (22 taxa; 42% of reported used parts), and were consumed at younger stages of growth. Fruit was the second most important plant part (16; 30%), and its importance in food ethnobotany is well-established among different communities. It is generally believed that the choice of fruit for consumption by locals is related to its peculiar or sweet taste [62]. It is important to mention that *Solanum americanum* contains toxic alkaloids [63], which are mainly found in its fruits [64], but nevertheless locals in the study area snack on a limited quantity of berries and they did not report any toxic effects. Fruits are primarily gathered by young community members and consumed on the spot. In the study area, both men and women are involved in gathering WFPs, which for most of the plants takes place from April to July. In general, the plants that are used raw as snacks are consumed by children. WFPs are gathered from mountains, forests, agricultural lands and home gardens during the spring and early summer, but the consumption of WFPs has significantly reduced in recent times. For the last two decades, political turmoil and military conflicts have greatly affected the socio-cultural and economic structure of local communities. Security instability has driven large numbers of people towards cities and thus local communities have become disconnected to agro-pastoral practices and their natural environment. Younger generations tend to move to cities to obtain an education and find a job as they do not desire the traditional way of life. Rapid social and economic evolution has interrupted the intergenerational transmission of ethnobotanical knowledge, even though elders still hold important ethnobotanical knowledge about WFPs. Moreover, some wild vegetables, such as *Chenopodium album, Malva neglecta, Rumex dentatus* and *Urtica dioica*, have been used in the past by the study communities but they have nowadays disappeared from the traditional food system.

Knowledge of wild food plant ingredients from traditional food systems may not only be linked to local biodiversity and the availability of plants, but also deeply embedded in everyday food practices, which are in turn highly variable and influenced by a complex combination of socio-cultural factors, such as the pervasiveness of industrialized food, food security status/socio-economic conditions, the importance of cultural identities and so on. Abbas et al. [18] reported that the traditional ecological knowledge (TEK) of WFPs in West Pakistan is partially eroded and that the majority of the reported species were quoted by approximately only one third of the informants. Research has also shown that TEK of WFPs is still alive in both the memory and the current practices of local people; for example, communities around Thakht-e-Sulaiman Hills, NW Pakistan [46], as well as the Hindu Kush mountains in North Pakistan [20], have reported important traditional knowledge of WFPs.

### 3.2. Cross-Cultural Comparison

**Figure 4:**
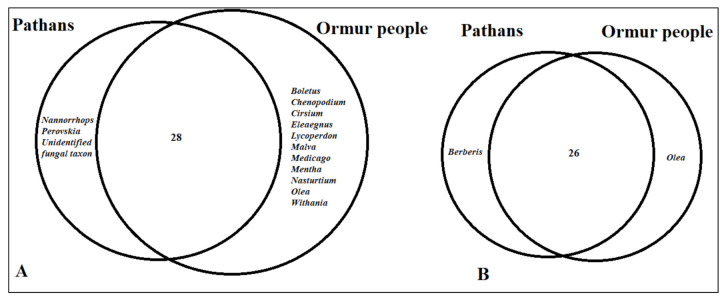
Venn diagram showing overlap of (**A**) all reported wild plant and fungal taxa (**B**) the most frequently reported wild plant and fungal taxa (quoted by more than 50% of informants) among the studied groups.

The food ethnobotanies of the two studied groups are presented in Venn diagrams (Figure 4). 

Comparative analysis indicates that three quarters of the WFP uses are commonly quoted among the two linguistic groups. Similarly, remarkable homogeneity was also found for frequently mentioned plant uses (quoted by more than 50% of informants). The commonalities among the food ethnobotanies of the two studied groups confirm some form of cross-cultural interaction among Pashtuns and Ormuri speakers, which shared the same socio-ecological space for centuries. The socio-cultural adaptations among Pashtuns and non-Pashtuns in North-West Pakistan were well-described by Barth [21]. The results here also highlight the socio-cultural negotiations among these ethnically diverse groups, which could be linked to symbiotic relationships and pluralism, as in case of Burusho, Kho, Shina and Wakhi peoples [19], and among Kalasha, Kho, Kamkatavari and Yidgha peoples in the Hindu Kush mountain range of Northern Pakistan [20]. Being an ancient diaspora, Ormur peoples slowly assimilated with the dominant Pashtun culture, and both groups were endogamic in the past, although they shared and share the same Sunni Islamic faith, but in the last few decades, there have been a large number of intermarriages between the groups, which may have been a possible factor behind the homogenization of their TEK. 

Pashtuns were historically pastoralists and the group retains crucial knowledge of WFPs found in pastures, such as *Berberis* spp., *Nannorrhops* spp. and *Perovskia* spp. With regard to the plant reports, both Ormuri speakers and Pashtuns show some divergences, exemplified by the former group, which exclusively quoted food uses for certain plant taxa (Figure 4). Ormuri speakers in fact gather more anthropogenic weeds than Pashtuns (see Figure 4), which may distinguish their foraging patterns and, more particularly, specify their possible horticultural-based human ecological origin. It is relevant to mention that most of the anthropogenic weeds reported among Ormur people were quoted by less than 50% of the participants. The implications of this finding may be crucial for understanding the possible dynamics of the Ormur cultural adaptation: it is possible that with their settlement in Kaniguram, the Ormuri speakers arrived with original foodscape patterns, and probably their assimilation into the dominant Pashtun culture eroded some unique plant uses. More importantly, Ormur ethnobotany has likely co-evolved and been supplemented with new WFP uses. We noted that some essential gastronomic perceptions, for instance tea prepared with the leaves of *Olea europaea*, are still very popular and preferred among the Ormur. Researchers have argued that the formation of certain consumption frameworks of WFPs are also shaped by sensory factors, i.e., preferences for specific tastes of a particular food plant [16]. It was interesting to learn that Pashtuns also used the leaves of *Olea europaea* for tea in the past. 

One of the Pashtun participants (62-year-old man) stated: “it was practiced in the old days, but now we buy tea from the local market. We don’t use the leaves of olive trees in tea as it goes against our pride to use the leaves of olive plants for tea in this modern time. It is the identity of Ormur speakers and no longer that of Pashtuns”. 

Such minor variation in processing and consumption methods may be related to the complex co-evolution in which both human ecological origins and sensory factors resulted in the manipulation of particular plant ingredients within the household [17]. We did not record any differences in processing and cooking methods of the wild vegetables between the two groups. 

Qualitative information on the ethnogenesis of the Ormur peoples is quite ambiguous and does not provide adequate evidence regarding their place of origin. As some aforementioned historical data on Ormuri speakers indicate that they originated either in Persia or Yemen, we compared those Ormuri plant names, which were different from the recorded Pashtun plant names, with Persian, Kurdish and Yemeni Arabic folk plant nomenclatures [49,50,51]. Comparative analysis revealed insightful similarities for certain taxa among the Ormuri, Persian and Kurdish phytonyms (Table 2). 

Of the 18 Ormuri idiosyncratic phytonyms we recorded, however, 8 are etymologically linked to Persian, 9 to Kurdish, 6 to Pashto, 1 to Yemeni Arabic (but probably indirect via Persian/Kurdish), while 4 have no link to the aforementioned languages. This could demonstrate that the Ormuri speakers did not originate in Yemen, but rather in Persia/Kurdistan, as also corroborated by the linguistic similarities of Ormuri with the North-Western Iranian group [23], i.e., the same family as Kurdish and some of the Iranian languages of Caucasus: the features in common with Pashto would be then due to successive contacts [67].

We observed also that the Ormuri plant nomenclature has been heavily affected by the plant nomenclature adopted by their next-door neighbors, the Pashtuns. We frequently found that all the Ormur study participants were able to name each plant in Pashto, but very few among them were able to name the same plants in the original Ormuri language. This phenomenon of language erosion may be due also to the fact that Ormur people adopted Pashto as “*lingua franca*” between them and their neighbors. During one interview, a study participant affirmed: “Military conflicts drove us towards cities which made it hard to maintain our language since we frequently interacted with Pashtuns. Our youngsters are losing their mother tongue and you are talking about naming wild food plants. Traditional knowledge is under threat because younger generations are not exposed to nature as we were and they don’t know much about wild food plants anymore, although elders still have the knowledge”. 

Morgenstierne [26] asserted that Ormuri speakers of both Kaniguram and Logar in Afghanistan have been significantly affected by their Pashtun neighbors and have freely borrowed numerous words from them. Kieffer [68] declared that the Ormuri language has reached the final stage of its resistance; it is used only in the home, and even there, due to exogamous marriages, its use is diminishing. 

### 3.3. Comparison with Pakistani Food Ethnobotany

On the basis of the comparative analysis presented in the previous section and the pre-existing literature on Pakistani food ethnobotany (see literature cited in the Data Analysis Section), we can outline some important uses that have significant relevance to the food ingredients we reported among the studied communities. As Pakistani ethnobotany has paid little attention to WFP uses, we thoroughly analyzed and compared the available studies with our research findings. Our Pakistani food ethnobotanical survey revealed three research studies which comprehensively gathered data on the food uses of wild taxa and reported significant traditional knowledge [19,20,46], while a couple of other studies partially focused on WFPs and recorded only wild vegetables [18,48]. Several research studies that focused on the importance of WFPs in traditional medicine were also taken into consideration for the comparison [42,43,44,45,47]. The majority of these studies were carried out in North-West Pakistan. In the country, a total of only three cross-cultural research studies, which includes our recent research [18,19,20], have been carried out among different linguistic and religious groups, illustrating that cross-cultural food ethnobotany is a newly established research area in Pakistan. The literature review demonstrates that the majority of the WFPs we recorded were also documented in previous food ethnobotanical studies, which included some frequently quoted taxa such as *Allium* spp., *Amaranthus* spp., *Berberis* spp., *Capparis* spp., *Caralluma* spp., *Chenopodium* spp., *Cotoneaster* spp., *Eremurus* spp., *Lepidium* spp., *Malva* spp., *Medicago* spp., *Mentha* spp., *Morchella* spp., *Portulaca* spp., *Rumex* spp., *Silene* spp., *Taraxacum* spp. and *Urtica* spp. Quantitative data extracted from the reviewed studies showed that the largest number of taxa were recorded from District Harnai, Balochistan (fifty-nine taxa), and these mainly focused on the ethnomedicinal values of wild vegetables [47]. A similarly remarkable number of plant taxa were also recorded from other areas of the country: fifty-eight taxa were reported from Chitral [20], fifty-five from District Kurram [18], fifty-one from Thakht-e-Sulaiman Hills [46], forty-five from Lesser Himalayas-Pakistan [42] and forty from District Ghizar, Gilgit-Baltistan [19]. The majority of these previously reported plants were frequently used as wild vegetables and raw snacks. The dominant families reported in these studies were Amaranthaceae, Apiaceae, Asteraceae, Brassicaceae, Fabaceae, Lamiaceae, Papilionoideae, Polygonaceae and Rosaceae. It is important to state that our review of the food ethnobotanical literature of Pakistan revealed, to the best of our knowledge, the use of a few taxa representing novel or rare wild food plant ingredients: *Cirsium arvense*, *Nannorrhops ritchiana*, *Oxalis corniculata*, *Periploca aphylla*, *Perovskia atriplicifolia*, *Viscum album* and *Withania coagulans* (Table 1). It is worth mentioning that our study recorded WFPs, i.e., *Oxalis corniculata* and *Withania coagulans*, which were used in lacto-fermentation, a food processing method never before reported in the ethnobotanical literature of Pakistan. 

### 3.4. Intellectual Property Rights (IPRs) over Traditional Ecological Knowledge: A Brief Lesson on Challenges from the Pakistani Case

Pakistan has been bestowed with an enormous body of TEK, and therefore it is imperative to safeguard its sovereignty and protect it from being misused in the patenting of non-original inventions. In the country, policy issues are extremely complex, making the subject of Intellectual Property Rights (IPRs) more difficult to interpret [69]. Pakistan is a signatory of many international agreements including the World Trade Organization (WTO) Agreement on Trade-Related Aspects of Intellectual Property Rights (TRIPs) [70], and the country became a party to the Nagoya Protocol on 21 February 2016 [71]. After Geographical Indications (GIs) came under the WTO Agreement on TRIPs, Pakistan started to develop a legal framework for the registration and protection of products and traditional skills developed within the country. The government formulated a set of legislative bodies that are responsible for implementing intellectual property rights. It is regrettable to outline that in the past, intellectual property laws were not effectively handled and interpreted in the country. One of the main obstacles was the lack of awareness regarding IPRs as well as laws among the general public [69]. According to Section 13 of the Intellectual Property Organization (IPO) of Pakistan Act, it is the responsibility of the state to spread awareness about IP problems via electronic and print media. Additionally, under Section 14, the authorities are responsible for developing IP via advocacy [72]. Mufti [73] pointed out that Pakistan is far behind in awareness on IPRs, where the lack of awareness is a key issue. It is worth mentioning that in the country, laws regarding patent copyright, trademarks and plant breeders’ rights are available, but laws protecting Geographical Indications and Genetic Resources, Traditional Knowledge and Folk Ware (GRTKF) have not yet been formulated [74]. Although a member of the Nagoya Protocol, the country has not effectively implemented recommendations directed by the Protocol. It has been claimed that the government is not paying sufficient attention to implementing the protocol, which is huge gap to be filled [69,75]. There is an urgent need to develop a system to preserve TEK and generate a system of TEK classification. It is also important to establish a Traditional Knowledge Digital Library (TKDL) with the goal of enhancing the quality of patent examination and allowing patent examiners to obtain access to pertinent information. In the country, national legislation should be reinforced to protect the TEK and IPRs of local communities to achieve the goal of the equity of benefits in resource consumption. Laws regarding disclosure and informed consent for any commercial use of TEK and genetic resources should be formulated as per recommendations of the Convention on Biological Diversity. 

## 4. Conclusions

This study recorded important TEK linked to WFPs within two cultural groups in Kaniguram, NW Pakistan. Plants were frequently consumed as snacks and wild vegetables. Aerial parts of plants and fruits were commonly used in food ethnobotanies. Three quarters of the recorded WFP uses were similar in the researched groups, but Ormuri speakers quoted a higher number of plant taxa and retained a deeper knowledge in terms of WFPs uses. Remarkable homogeneity was also observed for frequently reported WFP uses. The Ormur people used more anthropogenic weeds than Pashtuns, thus defining the former’s strong horticultural ecological origin. A comparative analysis with the ethnobotanical literature of Pakistan revealed the use of a few taxa representing novel or rare wild plant ingredients: *Cirsium arvense*,*Nannorrhops ritchiana*, *Oxalis corniculata*, *Periploca aphylla*, *Perovskia atriplicifolia*, *Viscum album* and *Withania coagulans.* Moreover, socio-cultural changes are posing serious threats to the permanence of TEK of the studied communities and especially that of Ormur people, which have undergone extensive cultural and linguistic adaptation to the dominant Pashtun culture. The current research may provide a baseline for future sustainable development programs aimed at designing bio-cultural safeguarding approaches in order to raise the public’s awareness of the importance of natural resources, including WFPs. It is also recommended that future studies carry out detailed syntheses by combining food ethnobotanical data with historical and archaeobotanical data in order to understand the processes of human adaptation and the spatial and temporal dynamics of plant food ingredients among different human settlements in uninvestigated geographical regions. 

## Figures and Tables

**Table 1:** Traditional uses of non-cultivated plants used among the studied communities in Kaniguram, South Waziristan (bold superscripts represent those uses quoted by more than 50% of the study participants within the two considered groups).

Botanical Taxon; Family; Botanical Voucher Specimen Code	Recorded Local Name	Parts Used	Recorded Local Food Uses	Ormur People	Pathans	Perceived Medicinal Uses (or Treated Illnesses)	Traditional Food Uses Previously Reported in Pakistan
*Allium* spp.; Amaryllidaceae	Zangli Yazza ^P^ Wozza ^O^ Khara Shabiay ^P^ Nanazrang ^O,P^	Aerial parts	^*^ Salad along with chapati bread ^O,P^	++	++		[19,20]
			* Onion are fried, seasoned and mixed with other vegetables ^O,P^	++	++		
*Allium jacquemontii Kunth;* Amaryllidaceae; SWAT000722	Yov-ree ^P^ Khukh ^O^	Aerial parts	* Raw snacks ^O,P^	++	++		[19,20]
			* Salad along with chapati bread ^O,P^	++	++		
*Amaranthus viridis* L.; Amaranthaceae; SWAT005470	Ranzaka ^P^ Sakaak ^O^	Aerial parts	Cooked: boiled and mixed with fried and seasoned onions ^O,P^	++	++	Vermicide ^P^, tonic ^P^	[18]
*Berberis calliobotrys* Bien. ex Koehne; Berberidaceae; SWAT000723	Kerai ^O,P^	Fruits	Raw snacks ^O,P^	-	++	Internal infections ^P^	[19,20]
*Caralluma tuberculata* N.E.Br.; Apocynaceae	Pamanai ^O,P^	Aerial parts	Cooked: boiled after mixing with fried and seasoned onions ^O,P^	++	++	Diabetes ^P^	[45]
*Celtis australis* L.; Cannabaceae; SWAT005474	Tarawan ^P^ Torawan ^P^ Tagh ^O^ Togha ^O^	Fruits	Raw snacks ^O,P^	++	++		[20]
*Chenopodium foliosum* Asch.; Amaranthaceae; SWAT005510	Khorrach ^P^	Fruits	Raw snacks ^O,P^	+	+		[20]
*Chenopodium album* L.; Amaranthaceae; SWAT005499	Kharrsaak ^O^	Aerial parts	* Cooked: Boiled after mixing with fried and seasoned onions ^O^	+	-		[19,20]
*Colchicum luteum* Baker; Colchicaceae; SWAT000724	Shamdai ^O,P^	Bulbs	Raw snacks ^O,P^	+	+		No
*Convolvulus arvensis* L.; Convolvulaceae; SWAT000725	Parwatiay ^O,P^	Leaves	* Cooked: boiled after mixing with fried and seasoned onions ^O,P^	+	+		[18]
*Cotoneaster microphyllus* Wall. ex Lindl.; Rosaceae; SWAT000726	Pashtawarki ^P^ Pashtawarkiay ^O^	Fruits	Raw snacks ^O,P^	++	++		[45]
*Cotoneaster racemiflorus* (Desf.) K.Koch; Rosaceae; SWAT000727	Sherawa ^P^ Sherowa ^O^	Fruits	Raw snacks ^O,P^	++	++		[45]
*Cirsium arvense* (L.) Scop.; Asteraceae; SWAT000728	Spiozir ^O^	Roots	* Raw snacks ^O^	+	-		No
*Elaeagnus angustifolia* L.; Elaeagnaceae; SWAT005808	Sanzala ^O^	Fruits	Raw snacks ^O^	++	-		[19,20]
*Ficus palmata* Forssk.; Moraceae; SWAT000729	Tugha ^P^ Inzir ^O^	Fruits	Raw snacks ^O,P^	++	++	Piles ^O^	[45]
*Lepidium draba* L.; Brassicaceae; SWAT000730	Bashka ^P^ Ghurghwast ^O^	Aerial parts	* Cooked: boiled after mixing with fried and seasoned onions ^O,P^	++	++		[18]
*Malva neglecta* Wallr.; Malvaceae; SWAT000731	Tikali ^P^ Isha-Tala ^P^ Techi^O^/Laska ^O^	Fruits	* Raw snacks ^O,P^	+	+	Root is ground and used as a sexual tonic ^O,P^	[18,45]
		Leaves	Cooked: boiled after mixing with fried and seasoned onions ^O^	+	-		
*Medicago monantha* (C.A.Mey.) Trautv.; Leguminosae; SWAT004745	Marghay Pasha ^P^ Batlakay ^O^	Aerial parts	* Raw snacks ^O,P^	+	+		[20]
			* Salad along with chapati bread ^O^	+	-		
*Mentha longifolia* (L.) L.; Lamiaceae; SWAT005790 and *M. spicata* L.; Lamiaceae; SWAT000732	Welani ^P^ Ghwan ^O^	Aerial parts	* Put inside dough to make bread ^O^	++	++	Gastric problems ^O,P^ (tea and salad)	[19,20]
			* Recreational tea ^O,P^	+	+		
*Morchella esculenta* (L.) Pers.; Morchellaceae; SWAT000733	Kurkeecho ^P^ Gurgeecho ^O^	Aerial parts	Cooked by mixing with fried and seasoned onions ^O,P^	++	++	Useful for removing phlegm ^O^	[19,20]
*Nannorrhops ritchieana* (Griff.) Aitch.; Arecaceae; SWAT000734	Mazari ^P^	Roots	* Snack in early stage ^P^	-	+		No
*Nasturtium officinale* R.Br.; Brassicaceae; SWAT005482	Dalamira ^P^ Tarmira ^O^	Aerial parts	* Salad along with chapati bread ^O,P^	++	++	Respiratory problems ^O^ (salad and cooked vegetables)	[20]
			* Cooked: boiled after mixing with fried and seasoned onions ^O^	+	-		
*Olea europaea* subsp. *cuspidata* (Wall. & G.Don) Cif.; Oleaceae; SWAT000735	Shawan ^P^ Shalwanai ^O^	Bark	* Recreational tea ^O^	++	-	Diabetes ^P^ (tea from leaves)	No
		Fruits	Raw snacks ^O,P^	++	++		[45]
		Leaves	Recreational tea ^O,^*^,P^	++	++		No
*Oxalis corniculata* L.; Oxalidaceae; SWAT000736	Tarweekai ^P^ Tuftufak ^O^	Aerial parts	* Raw snacks ^O,P^	++	++		No
			* Lacto fermentation ^O^: milk into yogurt	+	-		
*Periploca aphylla* Decne.; Apocynaceae; SWAT000737	Bararra ^O,P^	Latex	* Chewing gum ^O,P^	+	+		No
*Perovskia atriplicifolia* Benth.; Lamiaceae; SWAT000738	Sansubai ^P^	Flowers	* Raw snacks ^P^	-	+	Respiratory problems ^O,P^ (tea from flowers)	No
*Pinus gerardiana* Wall. ex D.Don; Pinaceae; SWAT004752	Zanrrghozai ^P^ Zoghak ^O^	Fruits	Cooked on a griddle ^O,P^	++	++		[20]
*Polygonatum verticillatum* (L.) All.; Asparagaceae; SWAT005969	Miralam ^O,P^	Aerial parts	* Cooked: boiled after mixing with fried and seasoned onions ^O,P^	+	+	Sexual tonic ^O,P^	[18]
*Portulaca quadrifida* L.; Portulacaceae; SWAT005970	Sormai ^P^ Vori ^O^	Aerial parts	* Cooked: boiled after mixing with fried and seasoned onions ^O,P^	++	++	Joint pain ^P^	[19,20]
*Quercus baloot* Griff.; Fagaceae; SWAT004748	Serray ^P^ Sat ^O^	Fruits	Cooked on a griddle ^O,P^	++	++	Gastric problems ^O^ (root decoction)	[20]
			Raw snacks ^O,P^	++	++		
*Quercus floribunda* Lindl. ex A.Camus; Fagaceae; SWAT000739	Serray ^P^ Sat ^O^	Fruits	Cooked on a griddle ^O,P^	++	++	Tonic^O^ (fruit)	[20]
			Raw snacks ^O,P^	++	++		
*Rumex dentatus* L.; Polygonaceae; SWAT005468	Zonda ^P^ Zando ^O^	Leaves	* Cooked: boiled after mixing with fried and seasoned onions ^O,P^	+	+		[19,20]
*Silene conoidea* L.; Caryophyllaceae; SWAT005514	Garrai ^O^ Kutkheelay ^P^	Seeds	* Raw snacks ^O,P^	+	+		[19,20]
*Sisymbrium irio* L.; Brassicaceae; SWAT005462	Zangali Charrsham ^P^ Saag ^P^ Khatakai ^O^	Leaves	* Salad ^O,P^	+	+		[20]
			* Cooked: boiled after mixing with fried and seasoned onions ^O,P^	+	+		
*Solanum americanum* Mill.; Solanaceae; SWAT005503, SWAT005803	Malgaibai ^P^ Khwazaibai ^P^ Garraibai ^P^ Cheekhruf ^O^	Fruits	Raw snacks ^O,P^	++	++		[20]
*Thymus linearis* Benth.; Lamiaceae; SWAT000740	Marvezay ^P^ Izbuk ^O^	Aerial parts	* Salad along with chapati bread ^O,P^	++	++	Cardiovascular problems ^O^ (salad and tea) Gastric problems ^P^ (salad and tea) Respiratory problems ^P^ (salad and tea)	[19]
			Recreational tea ^O,P^	++	++		
*Tragopogon gracilis* D.Don; Asteraceae; SWAT000741	Shabiay ^O,P^	Aerial parts	* Raw snacks ^O,P^	+	+		[45]
*Tulipa clusiana* DC.; Liliaceae; SWAT005973	Shamdai ^O,P^	Bulbs	Raw snacks ^O,P^	++	++		[19,20]
*Urtica dioica* L.; Urticaceae; SWAT005501	Dhur ^O^ Sezinkaiy ^P^	Aerial parts	* Cooked: boiled after mixing with fried and seasoned onions ^O,P^	+	+	Urinary tract problems ^O^	[19,20]
*Verbascum* sp.; Scrophulariaceae	Papakay ^P^ Zakhta ^P^	Leaves	* Raw snacks ^O,P^	+	+		-
*Viburnum cotinifolium* D. Don; Adoxaceae; SWAT000742	Margharrava ^P^ Margharrova ^O^	Fruits	Raw snacks ^O,P^	+	+		[45]
*Viscum album* L.; Santalaceae; SWAT000743	Warghosti ^O,P^		* Raw snacks ^O,P^	+	+		No
*Withania coagulans* (Stocks) Dunal; Solanaceae; SWAT000744	Shapianga ^O,P^	Fruits	* Lacto fermentation ^O^: milk into yogurt	+	-		No
*Boletus fraternus* Peck; Boletaceae	Kangal ^O^	Aerial parts	* Fried and seasoned along with onions ^O^	+	-		-
*Lycoperdon* sp.; Lycoperdaceae	Khomba ^O^	Aerial parts	* Fried and seasoned along with onions ^O^	+	-		-
Unidentified plant taxon	Makhlak ^P^ Makhilak ^O^	Aerial parts	* Cooked: boiled after mixing with fried and seasoned onions ^O,P^	++	++		-
Unidentified plant taxon	Kurashka ^O,P^	Leaves	* Recreational tea ^O,P^	++	++		-
Unidentified plant taxon	Kandola ^O,P^	Bulbs	* Raw snacks ^O,P^	+	+		-
Unidentified fungal taxon	Zangali Kadi ^P^	Aerial parts	* Fried and seasoned along with onions ^P^	-	++		-

^P^: Pashto; ^O^: Ormuri; *: wild food plants (WFPs) used in the past; -: not reported; +: quoted less than 50% informants; ++: quoted by more than 50% of informants.

**Table 2:** Comparative analysis of recorded Ormuri folk wild food plant names with folk plant nomenclature in Pashto, Persian, Kurdish and Yemeni Arabic (similar phytonyms are reported in bold).

Botanical Taxon	Ormuri Local Name	Pashto Local Name	Persian Local Names	Kurdish Local Names	Yemeni Arabic Local Names
*Amaranthus*	Sakaak	Ranzaka	Ashkeni, Barunak, Nadrou, Sorkhmaghz	Garisok, Gulzerin, Kilanshk, Koksor, Selmik	Dhadah, Ladah
*Celtis*	**Tagh, Togha**	Tarawan	Dadane, Daghdaghan, **Tagut**, Terakhte	Dakhum, Dargheni, Shengel, **Taug**, Tawin	**Tokah**^1^, Shergeb
*Chenopodium*	**Kharrsaak**	**Khorrach**	Poui, Salmak, Salmetareh, Teheghondarak	Peqake, Selmast	Maleh, Rakab-algamal, Ramram, Sala al-dunia, Shoqor-alhameer, Zerbeekh
*Cirsium*	Spiuzir ^2^	Aghzai ^3^	Kanghal, Shortikan, Tough	Karik, Kivar, Tiso	Shawk-hanash, Sanaf
*Ficus carica, F.cordata*	**Inzir**	Togha ^4^	**Andjir**, Kashgar, Tine	**Henchir**	Athab, Teen
*Lepidium draba*	**Ghurghwast ^5^**	**Bashka**	Bushi, **Garbast**, Mutcheh, Sondak	Dejnik, Kormik	Halqam
*Malva*	**Laska**, **Techi**	**Ishatala ^6^**, **Tikali ^7^**	Gurak, Kukar, Pachko, **Touleh**	Tolik	Khabiezeh, Khubez, Qarah
*Medicago*	Batlakay	Marghay pasha	Esbasse, Gan, Kollar	Ket, Shepel, Wenda	Fasfasah, Qadb
*Mentha*	Ghwan	Welani	None, Purchinak, Yarpuz	Nena, Piwin	Habaq, Naad
*Nasturtium*	**Tarmira ^8^**	**Dalamira**	Djerdjir, Koutine, Salmachu, **Torebak**	Bendik, Kuzele, **Teretiz**, Tumask	
*Oxalis*	**Tuftufak**	**Tarweekai ^9^**	Siahlak, **Torshak**	Sone, **Tirshok**	Hamadh
*Pinus*	**Zoghak**	**Zanrrghozai ^10^**	Nuje, Chalgos	Kai, Merkh, Sheinok	
*Portulaca*	Vori	Sormai	Khufa, Lunak, Mirri, Pichli, Shurdako	Perpine	Qalqalah, Raglah, Ragnah
*Quercus*	**Sat ^11^**	Serray	Golgan, Kereh, Mizi	Beru, Dedar, **Sek**	
*Silene*	**Garrai ^12^**	Kutkheelay	Dradir, Gharghar, Guto	**Giyarun**, Goshberkh, Halewek, Keshkul,	
*Sisymbrium*	**Khatakai**	Saag, Zangli charrsham	**Kakidj**, Shuvaran, Zarditi	Harik, **Khakshir**	Figel-algamal, Hurra
*Solanum americanum*	**Cheekhruf**	Garraibai, Khwazaibai, Malgaibai	Angurak, Kerezgui, Talkheh	**Chuchikan**, Hlerize, Tezle	Anamnam, Qumqam
*Thymus*	**Izbuk**	Marvezay	Avshan, **Azorbeh**, Khalvash, Oshom, Soter	Anikh, Hezwe, Ishan, **Zembur**	Saater

^1^ It could be a loanword from Persian or another Western Iranian language; ^2^ Possibly derived from Pashto *spay* meaning ‘dog’, maybe because of the thorny leaves, which make the plant difficult to be used; ^3^ From Pashto *aγzay* or *azγay* meaning ‘thorn’; ^4^ Similar to the Pashto phytonym for *Celtis*; ^5^ Possibly linked to the Persian *xār-/γār-* meaning ‘bush’ (De Chiara and Rossi [65]); ^6^ Possibly linked to Ormuri *laska* via metathesis; ^7^ Possibly linked to Ormuri *teči* and, by metathesis, with Persian *touleh* and Kurdish *tolik*; ^8^ The Ormuri and Pashto phytonyms are connected, possibly there is a link to the first part of the Persian and Kurdish phytonyms too; ^9^ In Persian, *torš* and Kurdish *tirš* mean ‘sour’; Persian *triw* has the same meaning: *toršak* and *tarwikay* are the equivalent; in an analogous way, Ormuri *tɔf* has the same meaning of ‘sour’, intensified by repetition, according to Morgenstierne [66]. The four modern Iranian languages then keep the same root, inherited from a proto-Iranian prototype; ^10^ Literally, *zaṇ-γozáy* ‘pine nuts’, with *γozá* ‘cone (pine)’ and *zúṇay* ‘seed’, the Ormuri word can be derived from or can be linked to Pashto with metathesis of *γ* and *z*. The Ormuri derivational form *-ak* corresponds to Pashsto *–ay*; ^11^ It seems possible a link between Ormuri *sat* and Kurdish *sek* due to the initial, even if the forms do not correspond exactly; ^12^ Interestingly, in Morgenstierne [66], the Ormuri word *gaṛäí* means ‘water-pot’ (corresponding to Waziri Pashto *gaṛei,* meaning ‘clay water pot’), and the shape of the *Silene* flowers resembles tiny water pots.

## Data Availability

All the data is provided in the article.
